# Developing theory-informed behaviour change interventions to implement evidence into practice: a systematic approach using the Theoretical Domains Framework

**DOI:** 10.1186/1748-5908-7-38

**Published:** 2012-04-24

**Authors:** Simon D French, Sally E Green, Denise A O’Connor, Joanne E McKenzie, Jill J Francis, Susan Michie, Rachelle Buchbinder, Peter Schattner, Neil Spike, Jeremy M Grimshaw

**Affiliations:** 1 School of Public Health and Preventive Medicine, Monash University, Level 6, The Alfred Centre, 99 Commercial Road, Melbourne, Victoria, 3004, Australia; 2 Primary Care Research Unit, University of Melbourne, 200 Berkeley St, Carlton, Victoria, 3010, Australia; 3 Health Services Research Unit, Health Sciences Building, University of Aberdeen, Aberdeen, AB25 2ZD , Scotland, UK; 4 Centre for Outcomes Research and Effectiveness, Department of Clinical, Educational and Health Psychology, University College London, WC1E 7HB, London, UK; 5 Monash Department of Clinical Epidemiology, Cabrini Hospital, Suite 41, Cabrini Medical Centre, 183 Wattletree Road, Malvern, VIC, 3144, Australia; 6 Department of General Practice, School of Primary Health Care, Monash University, Bldg 1, 270 Ferntree Gully Rd, Notting Hill, Victoria, 3168, Australia; 7 Clinical Epidemiology Program, Ottawa Health Research Institute, Ottawa, Canada; 8 Department of Medicine, University of Ottawa, 1053 Carling Avenue Administration Building, Room 2-017, Ottawa, ON, Canada, K1Y 4E9; 9 Department of Epidemiology and Preventive Medicine, School of Public Health and Preventive Medicine, Monash University, Level 6, The Alfred Centre, 99 Commercial Road, Melbourne, Victoria, Australia, 3004

## Abstract

**Background:**

There is little systematic operational guidance about how best to develop complex interventions to reduce the gap between practice and evidence. This article is one in a Series of articles documenting the development and use of the Theoretical Domains Framework (TDF) to advance the science of implementation research.

**Methods:**

The intervention was developed considering three main components: theory, evidence, and practical issues. We used a four-step approach, consisting of guiding questions, to direct the choice of the most appropriate components of an implementation intervention: Who needs to do what, differently? Using a theoretical framework, which barriers and enablers need to be addressed? Which intervention components (behaviour change techniques and mode(s) of delivery) could overcome the modifiable barriers and enhance the enablers? And how can behaviour change be measured and understood?

**Results:**

A complex implementation intervention was designed that aimed to improve acute low back pain management in primary care. We used the TDF to identify the barriers and enablers to the uptake of evidence into practice and to guide the choice of intervention components. These components were then combined into a cohesive intervention. The intervention was delivered via two facilitated interactive small group workshops. We also produced a DVD to distribute to all participants in the intervention group. We chose outcome measures in order to assess the mediating mechanisms of behaviour change.

**Conclusions:**

We have illustrated a four-step systematic method for developing an intervention designed to change clinical practice based on a theoretical framework. The method of development provides a systematic framework that could be used by others developing complex implementation interventions. While this framework should be iteratively adjusted and refined to suit other contexts and settings, we believe that the four-step process should be maintained as the primary framework to guide researchers through a comprehensive intervention development process.

## Background

Many resources have been directed toward improving the quality and safety of health care, but major problems persist [1]. Implementation interventions are interventions designed to change clinical practice behaviour and improve the uptake of evidence into practice. To date, implementation interventions have had limited and varied effects [2]. This may be due, in part, to a lack of explicit rationale for the intervention choice and the use of inappropriate methods to design the interventions [3-5].

The design of implementation interventions requires a systematic approach with a strong rationale for design and explicit reporting of the intervention development process [6-8]. One option is to use theory to inform the design of implementation interventions [3,9]. The UK Medical Research Council’s (MRC) guidance for developing complex interventions informed by theory [10-13] is useful as a general approach to designing an implementation intervention, but it does not provide detailed guidance about how to achieve this.

Multiple theories and frameworks of individual and organisational behaviour change exist, and often these theories have conceptually overlapping constructs [14-16]. Only a few of these theories have been tested in robust research in healthcare settings. There is currently no systematic basis for determining which among the various theories available predicts behaviour or behaviour change most precisely [17], or which is best suited to underpin implementation research [16,18]. Theories that have been used in previous implementation research include PRECEDE (Predisposing, Reinforcing, and Enabling Constructs in Educational Diagnosis and Evaluation), diffusion of innovations, information overload, and social marketing [5].

This article is one in a Series of articles documenting the development and use of the Theoretical Domains Framework (TDF) to advance the science of implementation research. The TDF was developed using an expert consensus process and validation to identify psychological and organisational theory relevant to health practitioner clinical behaviour change [19]. A set of 12 domains covering the main factors influencing practitioner clinical behaviour and behaviour change were identified: knowledge; skills; social/professional role and identity; beliefs about capabilities; beliefs about consequences; motivation and goals; memory, attention and decision processes; environmental context and resources; social influences; emotion; behavioural regulation; and nature of the behaviours. Relative to previously accepted, often implicit, models for developing interventions (for example, need to raise awareness, to provide information, to educate, need for an opinion leader or champion), these 12 domains provide an extensive framework that has greater coverage of potential barriers to change, and thus implies a greater range of potential intervention components.

Although improved health care can be facilitated at different levels of the health system, one important approach is to support individual health professionals to modify their clinical behaviour in response to evidence-based guidance [15]. The focus on this level is because much of health care is delivered in the context of an encounter between a health professional and a patient, making healthcare professional clinical behaviours an important proximal determinant of the quality of care that patients receive.

Development of implementation interventions can draw on theory, evidence, and practical issues in the following ways. Theory can be used to understand the factors that might influence the clinical behaviour change being targeted, to underpin possible techniques that could be used to change clinical behaviour [19], and to clarify how such techniques might work. Evidence can inform which clinical behaviours should be changed, and which potential behaviour change techniques and modes of delivery are likely to be effective. Practical issues then determine which behaviour change techniques are feasible with available resources, and which are likely to be acceptable in the relevant setting and to the targeted health professional group.

This paper outlines a method for developing an implementation intervention, drawing on the guidance from the UK MRC framework [10-12], and building on previously published methods for theory-informed intervention development [20,21]. We discuss how we used this method for developing an implementation intervention using the example of a recently developed intervention that we tested in the IMPLEMENT cluster randomised trial [22].

## Methods

### A method for developing implementation interventions to change clinical behaviour

We used a four-step approach, consisting of guiding questions to direct the choice of the most appropriate components of an implementation intervention (Table 1). The four steps represent: identifying the problem (who needs to do what, differently?); assessing the problem (using a theoretical framework, which barriers and enablers need to be addressed?); forming possible solutions (which intervention components could overcome the modifiable barriers and enhance the enablers?); and evaluating the selected intervention (how can behaviour change be measured and understood?).

**Table 1:** Steps for developing a theory-informed implementation intervention

**Step**	**Tasks**
STEP 1: Who needs to do what, differently?	· Identify the evidence-practice gap · Specify the behaviour change needed to reduce the evidence-practice gap · Specify the health professional group whose behaviour needs changing
STEP 2: Using a theoretical framework, which barriers and enablers need to be addressed?	· From the literature, and experience of the development team, select which theory(ies), or theoretical framework(s), are likely to inform the pathways of change · Use the chosen theory(ies), or framework, to identify the pathway(s) of change and the possible barriers and enablers to that pathway · Use qualitative and/or quantitative methods to identify barriers and enablers to behaviour change
STEP 3: Which intervention components (behaviour change techniques and mode(s) of delivery) could overcome the modifiable barriers and enhance the enablers?	· Use the chosen theory, or framework, to identify potential behaviour change techniques to overcome the barriers and enhance the enablers · Identify evidence to inform the selection of potential behaviour change techniques and modes of delivery · Identify what is likely to be feasible, locally relevant, and acceptable and combine identified components into an acceptable intervention that can be delivered
STEP 4: How can behaviour change be measured and understood?	· Identify mediators of change to investigate the proposed pathways of change · Select appropriate outcome measures · Determine feasibility of outcomes to be measured

Step 1. Who needs to do what, differently?

We selected the target clinical behaviours to be addressed, based on documented evidence-practice gaps. We specified the target behaviours in detail by asking the following questions: What is the clinical behaviour (or series of linked behaviours) that you will try to change? Who performs the behaviour(s)? And when and where do they perform the behaviour(s)?

Step 2. Using a theoretical framework, which barriers and enablers need to be addressed?

We chose a theoretical framework that we considered was most likely to inform the pathways of behaviour change. We used qualitative methods, underpinned by this theoretical framework, to identify the barriers and enablers to the pathways of change that were likely to influence the target clinical behaviours.

Step 3. Which intervention components (behaviour change techniques and mode(s) of delivery) could overcome the modifiable barriers and enhance the enablers?

Informed by our chosen theoretical framework, and empirical evidence about effectiveness of behaviour change techniques, we identified techniques to overcome the barriers and enhance the enablers. We first established the content of the intervention (what will actually be delivered), then we identified possible modes of delivery (how each chosen technique would be delivered) [23]. We based the final selection of behaviour change techniques and mode of delivery on what we considered was locally relevant, likely to be feasible, and could be implemented as a cohesive intervention.

Step 4. How can behaviour change be measured and understood?

We determined in advance the outcome measures for behaviour change and which mediators of change could be measured to evaluate the proposed pathways of change [24]. We based the selection of outcome measures on the availability of reliable and valid measures that were feasible to use.

## Results

The resultant IMPLEMENT intervention was delivered via two facilitated interactive small group workshops that were a combination of didactic lectures and small group discussions and activities. We also produced a DVD to distribute to all general practitioners (GPs) in the intervention group with the primary purpose of providing the material to those who could not attend the workshops. This alternative mode of delivering the same intervention content included film footage from the workshops and electronic resources related to acute low back pain management.

Step 1. Who needs to do what, differently?

The target behaviours for the IMPLEMENT intervention arose from two recommendations from the Australian evidence-based clinical practice guideline for acute low back pain [25]. The first target behaviour was to restrict the ordering of plain film x-rays to situations in which fracture is suspected because plain film x-rays are rarely helpful in the management of acute low back pain and are potentially harmful. The second target behaviour was to advise patients with acute non-specific low back pain to remain active because this reduces pain and disability.

We chose these target behaviours because they had strong supporting evidence, were potentially modifiable at a practitioner level, and were clinical behaviours to be performed by the GP during a clinical interaction early in the course of management of acute low back pain.

Step 2. Using a theoretical framework, which barriers and enablers need to be addressed?

To develop the IMPLEMENT intervention, we used the TDF [19] to identify the barriers and enablers to the target behaviours and to guide the choice of intervention components. Barriers to, and enablers of, the two target behaviours were identified in a qualitative study consisting of focus group interviews with 42 GPs in Victoria, Australia [26]. Each focus group was led by a trained facilitator who investigated the reasons GPs gave for practising, or not, in a manner consistent with the guideline recommendations. Questions were designed to explore the domains from the TDF for each of the two behaviours. Key domains were identified that described the specific barriers and enablers at a theoretical level, which then allowed us to access relevant evidence of likely effective behaviour change techniques.

Step 3. Which intervention components (behaviour change techniques and mode(s) of delivery) could overcome the modifiable barriers and enhance the enablers?

For the IMPLEMENT trial, our selection of behaviour change techniques was informed by a matrix that mapped behaviour change techniques to the theoretical domains, based on expert consensus about effectiveness for behaviour change [27]. We used the experience of the research team including clinicians and clinician educators, together with feedback from clinical colleagues on potential intervention approaches, to determine which behaviour change techniques and modes of delivery to select.

We chose the delivery mode of facilitated workshops because interactive education is familiar, acceptable, and feasible for GPs, and there is evidence that interventions delivered using this mode of delivery may change professional practice [28]. Also, this delivery mode could be linked to the requirements for Continuing Professional Development points for GPs in Australia. Finally, the intervention was assessed by the clinical members of the research team who checked that the proposed content was likely to be regarded by participants as relevant and helpful to their practice.

Table 2 provides details of the intervention development process of the IMPLEMENT intervention. The columns indicate how we linked specified barriers and enablers to theoretical domains and then identified behaviour change techniques and modes of delivery. For example, one identified barrier was lack of skill: some GPs reported that they lacked the communication skills to reassure patients that a plain film x-ray is unnecessary. We mapped this barrier to the domains ‘skills’ and ‘beliefs about capabilities’. It was considered that these domains were best addressed by using the behaviour change technique ‘rehearsal’ [27]. We delivered this in the facilitated workshop in a small group activity, where the GP took a clinical history with a trained simulated patient who repeatedly requested an x-ray, and the GP was asked to explain to the patient that a plain film x-ray was unnecessary, followed by a group discussion of the issues the GPs faced during the activity.

**Table 2:** Description of the steps used to choose the behaviour change techniques for the IMPLEMENT intervention

**Using a theoretical framework, which barriers and enablers need to be addressed? (Step 2)**	**Within which theoretical domains do the barriers and enablers operate?**	Which intervention components (behaviour change techniques and mode(s) of delivery) could overcome the modifiable barriers and enhance the enablers? (Technique; mode; content*) (Step 3)
Low awareness of the meanings and actions associated with the guideline’s key messages; low awareness of LBP red flags and skills in how to identify them	Knowledge (GP)	*Technique:* Information provision
		*Mode:* Facilitated workshop; GP opinion leader led; DVD
		*Content:* · GP opinion leader/content expert [29] presents information about the guideline key messages. Algorithm provided for diagnosis of red flags. Small group activity: participants reword key messages from the guideline to create behaviourally worded specific key messages (who, what, where, when) [30,31].
GPs’ perceptions of patients’ expectations and of patients’ beliefs about consequences	Knowledge (patient)	*Technique:* Information provision (directed at patient)
		*Mode:* Patient handout [32]
		*Content:* Handout contains lay language about key messages from the guideline [33]; GPs encouraged to give patients with acute LBP the handouts to reinforce verbal advice
Attitudes towards managing patients without x-ray, based on perceived consequences of the behaviour, e.g. fear of missing underlying pathology and belief that patient will feel reassured with an x-ray	1. Beliefs about consequences 2. Knowledge (GP)	*Techniques:* Information provision; Persuasive communication
		*Mode:* Facilitated workshop; DVD
		*Content:* · Highly respected senior clinician presents persuasive message about harms (harmful amounts of unnecessary radiation) and limited benefits (poor diagnostic utility) of x-ray for LBP · GPs provide examples of when important underlying pathology was missed due to absence of x-ray of LBP episode, giving opportunity for expert to discuss this case and demonstrate that x-ray wasn’t required.
Beliefs about negative consequences and beliefs about positive consequences of practising in a manner consistent with the guideline’s key messages	Beliefs about consequences	*Techniques:* Monitoring of consequences of own behaviour; Barrier identification; Persuasive communication
		*Mode:* Pre-workshop activity; facilitated workshop; DVD
		*Content:* · GPs record number of times they ordered plain x-ray and it didn’t change patient management, i.e. x-ray unnecessary. Highly respected senior clinician presents persuasive message about consequences of behaving in a manner consistent with the key messages.
Skills and beliefs about capabilities related to guideline key messages	1. Skills 2. Knowledge (GP) 3. Beliefs about capabilities	*Techniques:* Barrier identification; Model/demonstrate the behaviour; Rehearsal
		*Mode:* Facilitated workshop; DVD
		*Content:* · Participants write down wording of their last or usual message to stay active and then discuss in small groups. In pairs, with one GP role playing a patient with pre-prepared patient vignette, GP to create a script and role play with feedback from facilitator.
Perceived need to give the patient something to replace x-ray	Skills	*Techniques:* Provide instruction and modelling to increase a competing behaviour
		*Mode:* Facilitated workshop; DVD
		*Content:* Instruct, model/role-play and create a script to facilitate the competing behaviour of prescribing an activity log for patients (rather than giving x-ray referral).
Limited time to explain why patient does not need an x-ray and explain advice to stay active	Environmental context	*Techniques:* Information provision; Model/demonstrate the behaviour by a peer expert
		*Mode:* Facilitated workshop; DVD
		*Content:* use of handouts (patient handout [32] and activity log) to save time in consultation, and demonstration by a peer expert of how to incorporate into standard consultation.
Beliefs about the role of the GP when managing acute low back pain	Professional role and identity	*Techniques:* Persuasive communication; Provide opportunities for social comparison
		*Mode:* Facilitated workshop; DVD
		*Content:* · Highly respected senior clinician presents persuasive message about the role of the GP to minimise harm (from unnecessary irradiation from plain x-ray) and in encouraging patients to stay active. · Small group work discussion to allow opportunity for discussion of behaviours among peers.
Skills and beliefs about capabilities related to negotiating with/reassuring patients that plain x-ray is unnecessary	1. Skills 2. Beliefs about capabilities (in reassuring the patient that an x-ray isn’t helpful)	*Technique:* Rehearsal (prompt practice)
		*Mode:* Facilitated workshop
		*Content:* Small group activity: participants to take clinical history with a trained simulated patient to identify red flags. Simulated patients trained to expect and apply pressure for GP to order an x-ray. Discuss after task with feedback from facilitators [34-36].
GPs forget to give advice to stay active in standard consultation	Memory	*Technique:* Model/demonstrate the behaviour by a peer expert
		*Mode:* Facilitated workshop; DVD
		*Content:* Peer expert goes through 10 step management plan as a prompt for remembering CPG target behaviours
GPs’ perception that other people/organisations expectx-rays e.g., third party payors, radiologists	Social influences	*Techniques:* Information provision; Persuasive communication
		*Mode:* Facilitated workshop; DVD
		*Content:* Peer expert to discuss content of guideline and highlight organisations that endorse it.

* Technique: which behaviour change technique was chosen. Mode: how the technique was delivered. Content: what was delivered.

Detailed documentation of the full content of the IMPLEMENT facilitated workshops is available in the Additional file 1.

Step 4. How can behaviour change be measured and understood?

Table 3 outlines the constructs we planned to measure in the IMPLEMENT trial, describing outcomes measured to assess the causal pathway (mediating mechanisms of behaviour change), the practitioner outcomes and the patient outcomes.

**Table 3:** Mediators and outcomes measured in the IMPLEMENT trial

**What to measure**	**Measures**
Mediating mechanisms of behaviour change	· Constructs theorised to be mediators of behaviour change (measured by practitioner survey)
Practitioner outcomes	· X-ray referral rates (measured by patient file audit) · Patient given advice to stay active (measured via patient survey)
Patient health outcomes	· Low back pain outcome measures (pain and disability measured via patient interview)

## Discussion

We have illustrated a four-step method for developing an intervention designed to change clinical behaviour based on a theoretical framework. We have demonstrated the use of this method in a case study of designing the IMPLEMENT intervention, an intervention designed to improve the management of low back pain in general practice [22].

Many researchers who assess barriers and enablers for implementation problem assessment do not do this within a theoretical framework and are therefore limited to pragmatic, rather than theoretically informed, solutions [37]. The use of our method when designing implementation interventions allows the use of theory and empirical research, along with the results of mixed methods research, to decide upon intervention components and to build a complex intervention. This will allow for further exploration of the associations between intervention components and intervention effects [38]. We also encourage researchers involved in developing implementation interventions to better document and report their own development process thus generating a body of knowledge about explicit methods for developing these interventions.

### Strengths of this method

The main strength of this four-step method is that it can be used as a guide for implementation intervention developers through a systematic method of moving from target behaviours, to theoretical domains, to behaviour changes techniques, and finally a full implementation intervention. By basing implementation interventions on a theoretical approach to behaviour change and linking this to relevant and effective behaviour change techniques, researchers can make explicit, and thus investigate, the hypothesised mechanisms of change. We propose a streamlined approach moving directly from identified theoretical domains relevant to the implementation problem to behaviour change techniques.

Multiple theories and frameworks of individual and organisational behaviour change exist [14-16], and choosing an appropriate theory for designing implementation interventions is challenging [16-18]. Setting predetermined criteria for selection of theories may assist, for example, including consideration of the evidence base for the theory, the relevance to the setting, and perceived usefulness of the theory. Also, using a broadly based theoretical framework for behaviour change, rather than a single theory, may allow a more comprehensive examination of potential barriers and enablers, and possible mechanisms linking them to the target clinical behaviour. The TDF is arguably the most comprehensive framework for designing implementation interventions as it offers broad coverage of potential change pathways; however other theoretical frameworks, or specific theories, could be used.

There are many potential delivery modes in the clinical setting for most behaviour change techniques [27], including educational meetings, educational detailing, reminder systems, and audit and feedback [39]. We suggest the choice of the delivery mode is guided by local context and what is acceptable and feasible in the target group.

### Potential limitations of this method

There is subjectivity in this proposed process of designing implementation interventions of combining research evidence, matrix mapping, and feasibility information. Further empirical work is required to test the method in different contexts and examine the links between theoretical assessment and behaviour change techniques. There are alternative ways of operationalising this method. For example, in other studies the TDF has been used to identify key domains followed by predictive theory-based surveys to check whether the domains regarded as ‘key’ actually do predict clinical practice [40]. Another approach has been to use the TDF to identify specific theories of behaviour change to inform the intervention [40-42]. Documentation and careful reporting of the development process of implementation interventions will further this field.

In IMPLEMENT, barriers and enablers were assessed by conducting focus groups with GPs. Hence, the intervention developed was directed at the individual clinician. However, in broader application of the TDF, although data may be gathered at an individual level, theory is not restricted to the individual level and may address organisational determinants of behaviour change, such as the included domains Environmental context and resources, Professional role and identity, and Social influences which address issues beyond the individual clinician.

This method of intervention development requires considerable time and resources. Developing the IMPLEMENT intervention involved time of a PhD student (SDF), his supervisors (SEG and RB), and input from the rest of the research team via teleconferences and email correspondence over 12 months. During this time we were also developing the method itself, adding to the time needed. Full detail of the resources required to develop the IMPLEMENT intervention will be reported in a separate economic evaluation publication.

The resources required could be seen as a necessary component of the development of complex behaviour change implementation interventions [12]. Resources to enable this will depend on funding models allocating sufficient funds for the intervention development component of research studies and to require this as a component of guideline development and implementation initiatives. Currently, many funding bodies expect that, at the time at which funding applications are submitted, the intervention protocol for complex interventions is already well formulated [43]. This contrasts with, for example, pharmacologic interventions, for which it is accepted that a decade or two of basic research is required before an intervention is ready for trialling. The publication of the new guidance for the MRC framework [12], along with applications of the framework which demonstrate the resource intensive nature of this work, will hopefully facilitate increased funding for this important activity, increasing the likelihood that interventions will be fit for evaluation in trials, and will therefore better utilise research investments.

## Conclusions

We have illustrated a four-step systematic method for developing an intervention designed to change clinician behaviour based on a theoretical framework. We propose a streamlined approach moving directly from identified theoretical domains relevant to the implementation problem to behaviour change techniques. This method is a conceptual aid, rather than a rigid prescription; it may be iteratively adjusted and refined to suit other contexts and settings. The process outlined here can be used by researchers and quality improvement practitioners to guide a comprehensive intervention development process.

By basing implementation interventions on a theoretical approach to behaviour change and linking this to relevant and effective behaviour change techniques, researchers can make explicit, and thus investigate, the hypothesised mechanisms of change. We have argued that using such a method can facilitate the development of theory-informed implementation interventions. Finally, appropriate reporting of the processes used to develop the interventions, and of the components of the intervention, is necessary.

## Competing interests

Denise O’Connor and Susan Michie are Associate Editors for Implementation Science and Sally Green and Jeremy Grimshaw are members of the editorial board. All decisions on this manuscript were made by another editor. All other authors declare that they have no competing interests.

## Authors’ contributions

SF wrote the first draft of this manuscript with comments from SG, RB, SM, and JG, and then the remaining authors. All authors contributed to the ideas in this paper and SF acts as guarantor. All authors read and approved the final manuscript.

## Supplementary Material

Additional file 1 Detailed description of IMPLEMENT intervention. Click here for file
