# Supplementary material for: Developing theory-informed behaviour change interventions to implement evidence into practice: a systematic approach using the Theoretical Domains Framework
Source: Implement Sci. 2012 Apr 24;7:38. doi: 10.1186/1748-5908-7-38 (PMC3443064; doi:10.1186/1748-5908-7-38)
Supplement: Additional file 1 — Detailed description of IMPLEMENT intervention. [file 1748-5908-7-38-S1.pdf]

Additional File: Detailed description of IMPLEMENT intervention

The management of acute low-back pain in general practice

Table of Contents:

1. IMPLEMENT intervention Program Overview.. 2
2. IMPLEMENT intervention Detailed Program 3
3. Small group work No. 1 – discussion of pre-workshop activity about x-ray.......... 5
4. Small group work No. 2 – Making recommendations behaviourally specific........ 6
5. Small group work No. 3 – Red flag screening practical (possible scenarios)........ 9
6. Small group work No. 4 – Discussion of pre-workshop activity about advice to stay active 12
7. Small group No. 5: Talking with patients: putting recommendations into practice 13
8. Action planning 15
9. Pre-workshop activity 16
10. Post-workshop activity 18
11. Red flag screening algorithm 20
12. Activity log 22
13. Patient handout 24

1. IMPLEMENT intervention Program Overview

Session I – Confidence in Diagnosis

Item	Format and content	Time (evening sessions)	Time (day sessions)
1	Food	6.00pm	10.00am
2	Welcome and Introductions	6.15pm	10.15am
3	Small group work No. 1	6.35pm	10.35am
4	Acute LBP guideline	7.15pm	11.15am
5	Small group work No. 2	7.30pm	11.30am
6	Break	7.45pm	11.45am
7	Plain film x-ray for acute LBP	8.00pm	12.00pm
8	History and Examination	8.15pm	12.15pm
9	Small group No. 3	8.30pm	12.30pm
10	Summary	9.20pm	1.20pm
11	Close	9.30pm	1.30pm

Session II – Intervention

Item	Format and content	Time (evening sessions)	Time (day sessions)
1	Food	6.00pm	1.30pm
2	Welcome and Introduction to Session II	6.15pm	1.45pm
3	Small group work No. 4	6.30pm	2.00pm
4	Update on what works and what doesn’t	7.00pm	2.30pm
5	Advice to stay active recommendation	7.20pm	2.50pm
6	Break	7.30pm	3.00pm
7	10 steps of clinical management as a framework for LBP management	7.45pm	3.15pm
8	Small group No. 5	8.15pm	3.45pm
9	Summary	9.00pm	4.30pm
10	Action planning	9.10pm	4.40pm
11	Close	9.30pm	5.00pm

2. IMPLEMENT intervention Detailed Program.

Session I – Confidence in Diagnosis

Time	Title	Content
10.00am	Coffee and tea	
10.15am	Welcome and Introductions	• Introductions of investigators, facilitator<br>• Ice breaker, for example WorkCover video trivia question about it<br>• Set ground rules<br>• Agenda and content for session
10.35am	Small group work No. 1<br><br>Discussion of pre-workshop activity about x-ray	• Discussion in small groups (3-4) with flip charts and fed back by appointed GP to larger group<br>• 5 minutes at end of this section for Facilitator to note-take barriers and enablers on whiteboard/flip chart and revisit throughout session using persuasive communication techniques
11.15am	Guideline recommendations about diagnosis and x-ray use	• Didactic presentation<br>• Introduction to LBP<br>• Guideline development and stakeholders<br>• Recommendations about making a diagnosis
11.30am	Small group work No. 2<br>Making recommendations behaviourally specific	Using NICS tool - by who, applying to who, what, where, when
11.45am	Coffee and tea	
12.00pm	Plain film x-ray for acute LBP	Harms of x-ray<br>Utility of x-ray<br>Lead in to utility of red flag screening
12.15pm	History and Examination	Presentation and demonstration (with simulated patient) of identifying red flags
12.30pm	Small group No. 3<br>Patient assessment practical	Various scenarios using simulated patients
1.20pm	Summary	Facilitator to lead discussion:<br>• Reflect on barriers on whiteboard<br>• Questions<br>• Outstanding issues<br>• Quick intro to Session 2

Session II – Intervention: "move it or lose it"

Time	Title	Content
1.30pm	Lunch	
1.45pm	Welcome and Introductions	• Introduce themselves – 1st name, ice breaker - WorkCover video<br>• Recap on last session<br>• Ground rules, agenda for session
2.00pm	Small group work No. 4<br>Discussion of pre-workshop activity about advice to stay active	• Discussion in small groups (3-4) with flip charts and fed back by appointed GP to larger group<br>• Facilitator/scribe to note-take barriers and enablers on whiteboard and revisit throughout session using persuasive communication techniques
2.30pm	Update on what works and what doesn't	• Didactic presentation<br>• Guideline recommendations for interventions
2.50pm	Making recommendations behaviourally specific for advice to stay active	• Whole group brainstorm<br>• Using NICS tool - by who, applying to who, what, where, when
3.00pm	Coffee and tea	
3.15pm	10 steps of clinical management as a framework for LBP Management	• Didactic presentation (facilitator - if familiar with framework) with LBP relevant examples
3.45pm	Small group No. 5<br>Talking with patients: Putting recommendations into practice	Referring to pre-prepared scripts, participants practice with a partner and create scripts for themselves about the two key messages that are workable, time efficient, reinforces patient education and adhere to principles of negotiation.<br><br>Give each pair two different scenarios of approximately 10 minutes each
4.30pm	Quick Summary	Reflect on barriers (persuasive communication)<br>Questions?<br>Outstanding issues?
4.40pm	Action planning	• 3 min didactic blurb on evidence for action planning<br>• If situation Y is encountered then I will initiate behaviour X<br>• These are situations where the action plan will be useful; attention to detail

3. Small group work No. 1

Discussion of pre-workshop activity about x-ray.

Please refer to the pre-workshop activity document.

In pairs or groups of three, please discuss your pre-workshop activity (the x-ray section).

Some questions to consider:

- What were the major issues you had?
- Do you think you have practised in a way that is consistent with the guideline recommendation (regarding the key message that diagnostic x-rays are rarely necessary in the management of acute low-back pain)?
- Were there any difficulties in implementing this key message?

Notes:

4. Small group work No. 2

Making recommendations behaviourally specific

(Adapted from "Making recommendations actionable", National Institute of Clinical Studies, 2006").

Many methods can be used in an effort to increase the implementation of guidelines. One of the simplest is to be as specific as possible when forming guideline recommendations. People who intend to change their behaviour are more likely to do so if they have a specific behavioural plan (a plan for exactly what to do and when to do it) (Gollwitzer 1999). The clearer and more detailed the plan the more likely it is to be followed.

Benefits of rewriting recommendations in a clear and specific style include:

- Greater clarity about what needs to be done
- Greater certainty about whether appropriate action has been taken
- Easier development of benchmarks or performance indicators (Michie 2005)

Translating recommendations into active verbs (ie "should" rather than "may") can reveal areas where actions to be taken are not clear (Michie 2004).

To increase the chance of successful implementation of guidelines, recommendation should be rewritten in a way that makes them concrete and behaviourally specific. They should clearly spell out what to do, and in which situation (Grol 1998 & Shiffman 2005). In order to do this, try specifying things such as:

- What (ie what needs to be done)
- How (ie how it should be done)
- Who (ie who should do it)
- When (ie in which situation)
- Where (ie in which patient group)
- Why (ie why it should be done)

Of course, it is important to remember that there should still be some flexibility to allow for clinical judgement when needed.

Example:
The following are recommendations from a NICE guideline about managing schizophrenia. The recommendations were adapted to make them more actionable (Michie 2004):

Original recommendation	Derived local recommendation
Cognitive behavioural therapy (CBT) should be available as a treatment option for people with schizophrenia	Offer cognitive behavioural therapy to everyone with schizophrenia
Acute day hospitals should be considered as a clinical and cost effective option for the provision of acute care, both as an alternative to acute admission to inpatient care and to facilitate early discharge from inpatient care	Offer acute day hospital treatment to inpatients or those facing acute admission to inpatient care

References
Gollwitzer PM (1999). Implementation intentions: Strong effects of simple plans. American Psychologist 54(7): 493-503

Grol R, Dalhuijsen J, Thomas S, Veld Cit, Rutten G & Mokkink H (1998). Attributes of clinical guidelines that influence use of guidelines in general practice: observational study. BMJ 317(7162): 858-61
Michie S & Lester K (2005a). Words matter: increasing the implementation of clinical guidelines. Qual Saf Health Care 14(5): 367-70
Michie S & Johnston M (2004). Changing clinical behaviour by making guidelines specific. BMJ 328(7435): 343-5
Shiffman RN, Dixon J, Brandt C, Essaihi A, Hsiao A, Michel G & O'Connell R (2005). The GuideLine Implementability Appraisal (GLIA): development of an instrument to identify obstacles to guideline implementation. BMC Med Inform Decis Mak 5: 23

Notes:

Workshop
In pairs or groups of three, modify the recommendations from the acute low-back pain guideline using the NICS format (What, How, Who, When, Where, Why).

Original recommendation #1
Plain x-rays of the lumbar spine are not routinely recommended in acute non-specific low back pain as they are of limited diagnostic value and no benefits in physical function, pain or disability are observed.

Your version of the recommendation…

____

____

____

Original recommendation #2
Advice to stay active provides a small beneficial effect on pain, rate of recovery and function compared to bed rest and compared to a specific exercise regime in mixed (acute/chronic) populations with low back pain.

Your version of the recommendation…

____

____

____

Original recommendation #3
Advice to stay active reduces sick leave compared to bed rest in mixed populations with low back pain.

Your version of the recommendation…

____

____

____

5. Small group work No. 3

Patient assessment practical

In groups of two to three, conduct a history of a simulated patient exploring the different scenarios presented.

Notes:

Possible Scenarios:

Scenario 1: Severe low back pain
Pseudo-name of SP: Jill Wilson; aged between 20-30 years old; office worker
Chief complaint: low back pain
Demeanour: obviously in pain and anxious (that has a serious condition).

History of symptoms
Onset: No specific incident.
Type of discomfort: Pain varies in intensity throughout the day, mostly 9 out of 10.
Location: Lower back region (below the ribs and above the level of the waist/buttocks)
Radiation: None.
Duration: Low back pain began 1 week ago.
Relieving factors: Lying on back with knees bent, nurofen
Aggravating factors: Bending, after sitting.
Associated symptoms: None.
Health history: No other health concerns. No previous history of low back pain.

Scenario 2: Persistent low back pain
Pseudo-name of SP: Kylie Withers or Scott Barrow, aged between 30-45 years old; council worker
Chief complaint: Low back pain that is not improving after five weeks.
Demeanour: The patient has consulted this GP (or others in the practice) three to four times over the course of this episode of low-back pain. The pain has not improved, and patient is now concerned that not enough is being done by the doctors. The patient's work and normal activities have been significantly reduced since the onset of the pain. The patient thinks that an x-ray is required to "find out what is wrong".

History of symptoms
Onset: No specific incident.
Type of discomfort: Pain varies in intensity throughout the day, mostly 7 out of 10.
Location: Lower back region (below the ribs and above the level of the waist/buttocks)
Radiation: None.
Duration: Low back pain began 6 weeks ago.
Relieving factors: Lying on back with knees bent, heat.
Aggravating factors: Bending, standing after sitting.
Associated symptoms: None.
Health history: No other health concerns. No previous history of low back pain.

Scenario 3: Low back pain with an aggressive patient
Pseudo-name of SP: David Harding, a between 30-50 years old; office worker.
Chief complaint: Low back pain
Demeanour: Aggressive. Frustrated with the pain and insistent that he requires an x-ray.

History of symptoms
Duration: Pain has been present for six weeks.
Onset: The pain started two days after moving heavy furniture at home.
Type of discomfort: Mild ache.
Location: Lower back region (below the ribs and above the level of the waist/buttocks)
Radiation: None.
Relieving factors: Massage, anti-inflammatory medication.
Aggravating factors: Lifting heavy objects.
Associated symptoms: None. Otherwise well.

Health history: No other health concerns. Has had previous, similar episodes of low back pain that have lasted one to two weeks.

Scenario 4: Low back pain patient insistent on an x-ray
Pseudo-name of SP: Matthew McMahon or Joanne Hatty, aged between 20 and 50.
Chief complaint: Low back pain
Demeanour: Patient is insistent on an x-ray and during the consultation tells GP that they will go somewhere else if they don't order one.

History of symptoms
Onset: No specific incident.
Type of discomfort: Pain varies in intensity throughout the day, mostly 5-6 out of 10.
Location: Lower back region (below the ribs and above the level of the waist/buttocks)
Radiation: None.
Duration: Low back pain began 3 weeks ago.
Relieving factors: Lying on back with knees bent, heat.
Aggravating factors: Bending, standing after sitting.
Associated symptoms: None.
Health history: No other health concerns. No previous history of low back pain.

6. Small group work No. 4

Discussion of pre-workshop activity about advice to stay active

Please refer to the pre-workshop activity document.

In pairs or groups of three, please discuss your pre-workshop activity (the giving of advice to stay active section).

Some questions to consider:

- What were the major issues you had?
- Do you think you have practised in a way that is consistent with the guideline recommendations (regarding the key message that remaining active reduces pain and disability)?
- Were there any difficulties in implementing this key message?

Notes:

____

____

____

____

____

____

Discussion of pre-workshop activity about advice to stay active

7. Small group work No. 5

Talking with patients: putting recommendations into practice

The aim of this small group activity is to bring all previous information discussed in these two sessions together. Using the 10 step management plan, you will role play (with another GP) a “typical” consultation with patient with acute low-back pain, ie take a history, perform a quick examination and implement a management plan, taking into account the needs of the patient presented.

Each pair will be given two different scenarios. This will give each of you the opportunity to undertake the role of the patient and the role of the GP.

Scenarios will include the following:

- GP pushed for time
- Anxious patient (about potential for serious disease)
- Patient with co-morbidities
- Patient with severe low-back pain
- Patient who is not responding to your previous treatment
- A patient with recurrent low-back pain
- Patients with varying baseline activity levels (eg athlete vs couch potato)
- Losing the patient to another provider
- Fears about consequences (of staying active)
- Patient may think GP is unsympathetic to their pain
- Dealing with patient insistent for an x-ray

The **10 step management plan** is:

1. Tell the patient the diagnosis
2. Establish patient’s knowledge
3. Establish patient’s attitude
4. Educate the patient
5. Develop a joint management plan for the presenting problem
6. Explore other preventive opportunities
7. Reinforce involving the patient (and others)
8. Provide take away information
9. Evaluate the consultation
10. Arrange follow-up

One possibility that you may want to explore is **creating your own script**. A good script saves you time and with practice becomes second nature when managing patients with acute low-back pain.

The main aspects to incorporate into a script include the following:

- Provide reassurance:
  - nothing dangerous
  - expect recovery soon
- Give advice to stay active (including activity log)
- Give patient handout
- Symptomatic measures if required, eg simple analgesics, local heat

Notes:

8. ACTION PLANNING

There is a body of evidence about the effects of "if-then" planning on goal achievement, that is, making a specific plan for what to do in a particular circumstance to help you to reach your goal. For example, if your goal is to achieve your CME points for these workshops, your "if-then" plan might be "*if* I receive mail from the IMPLEMENT study team, *then* I will not throw it in the bin". A meta-analysis by Gollwitzer and Sheeran (2006) showed that if-then planning substantially increased the likelihood of a person reaching their goal.

Your plan should be precise (describe how you will act in a specific situation) and feasible (the situation is likely to occur and the action is achievable).

Please write down some things that you will do as a result of attending these workshops – and refer to them frequently over the next few weeks!

List 3 things you've learnt

1. ____

2. ____

3. ____

List 3 things you want to follow up from these workshops

1. ____

2. ____

3. ____

List 3 things you'll do when you get back to your organisation

1. ____

2. ____

3. ____

Reference:

Gollwitzer PM & Sheeran P (2006). Implementation intentions and goal achievement: a meta-analysis of effects and processes. In: Zanna, MP. Advances in experimental social psychology. US, Academic Press: 69-119

9. Pre-workshop activity

Dear Dr,

These workshops and the associated activities attract continuing education points (RACGP 30 Category 1 points; ACRRM 6.5 PDP points). In order for you to be allocated these points you must attend both sessions and complete a pre-workshop activity (this one), a post-workshop activity and a workshop evaluation. Please complete this pre-workshop activity and bring it along with you to the workshops, or return to the address below. A post-workshop activity will be forwarded to you for completion and return to us, approximately 4 weeks after the completion of the workshops.

Name: Dr _______________ QA&CPD/ACRRM number: _________
Please modify if incorrect

Please have a think about the following questions and write down answers which are relevant to your management of patients in your practice. All the questions refer to patients with acute (less than 3 months) non-specific low-back pain. The answers to these questions are for your reflection.

Question 1:
Approximately how many patients do you believe you would treat for acute low-back pain in an average month?

Answer: __________________________________

Question 2:
I am familiar with the NHMRC-endorsed clinical practice guidelines for acute low-back pain.
☐ Yes ☐ No

If yes, I use the Guidelines in my daily management of acute low-back pain (please circle the appropriate response).

1	2	3	4	5
Never		Sometimes		All the time

Question 3:
How would you rate your confidence to manage a patient with acute low back pain (please circle the appropriate response)?

1	2	3	4	5
Not at all confident		Somewhat confident		Very confident

Question 4:
Please indicate which areas of the clinical guidelines in the management of acute non-specific low-back pain you would like to know more about:

- ☐ Evidence for the Guidelines
- ☐ Appropriate low-back pain history and examination
- ☐ Diagnostic triage
- ☐ Role of radiography
- ☐ Role of bed rest
- ☐ Role of giving advice to stay active
- ☐ Role of other therapies

Are there other areas that you would like covered in an educational activity on this subject?

Activity

This activity will be discussed at the workshops. Please consider the questions beforehand so you will gain most from the educational workshops.

Please identify up to 10 acute low-back patients that have consulted you recently. Over the course of your management of each of these patients, please consider the following:

Part A. About using imaging

a. Did the patient ask for an x-ray?

b. Did you refer the patient for a plain film x-ray? What were your reasons for referring for an x-ray or not?

c. What were the consequences of your decision?

d. Did the consequences impact on how you treated the patient?

Part B. About your advice

a. Did you advise this patient to stay active?

b. What were your reasons for this?

c. What were the consequences of this decision?

d. Did the patient articulate a concern about their activity?

Part C. Patient education materials

If you use any patient education materials in your practice for the management of acute low-back pain, please bring them along to the workshops.

10. Post-workshop activity

Dear Dr ___________,

We hope you found the workshops enjoyable and of value. Prior to the workshops, you completed a pre-workshop activity. To complete the requirements for you to be allocated the full complement of continuing education points for this activity (RACGP 30 Category 1 points; ACRRM 6.5 PDP points), please complete the following post-workshop activity. You need to complete this activity four weeks after you attended the second workshop. We will send you a reminder approximately 4 weeks after the completion of the workshops.

Name: Dr _________________________ QA&CPD/ACRRM number: _________

Please have a think about the following questions and write down answers which are relevant to your management of patients in your practice. All the questions refer to patients with acute (less than 3 months) non-specific low-back pain. The answers to these questions are for your reflection.

Question 1:
Approximately how many patients do you believe you have treated for acute low-back pain in the last one month?

Answer: ______________

Question 2:
I am familiar with the NHMRC-endorsed clinical practice guidelines for acute low-back pain.
☐ Yes ☐ No

If yes, I use the Guidelines in my daily management of acute low-back pain (please circle the appropriate response).

1	2	3	4	5
Never		Sometimes		All the time

Question 3:
How would you rate your confidence to manage a patient with acute low back pain (please circle the appropriate response)?

1	2	3	4	5
Not at all confident		Somewhat confident		Very confident

Question 4:
Approximately what proportion of patients with acute non-specific low-back pain do you remember referring for an x-ray?

Answer: __________________

Question 5:
Approximately what proportion of patients with acute non-specific low-back pain d [illegible] giving the advice to “stay active”?

Please turn over

Answer: ________________________________

Question 6:
What does ‘advising these patients to stay active’ mean to you?
 advising the patient not to lie in bed
 advising the patient to do specific back exercises
 advising the patient to do general exercises (eg. walking)
 advising the patient to continue with their normal daily activities within the limits of pain
 other (please specify)
____

Question 7:
Please indicate which areas of the clinical guidelines in the management of acute non-specific low-back pain you need to know more about:

□ Evidence for the Guidelines
□ Diagnostic triage
□ Role of radiography
□ Role of bed rest
□ Role of giving advice to stay active
□ Role of analgesics
□ Role of other therapies
□ Appropriate low-back pain history and examination

Are there other areas that you would like covered in an educational activity on this subject?

____

____

11. Red flag screening algorithm

Management of acute low-back pain in General Practice

Acute (<3 months) low-back pain confirmed?

NO → EXIT: An alternative management plan specific to the condition is required

YES ↓

Is there a spinal cord or cauda equina lesion?

YES → EXIT: Emergency Referral to a Spinal Surgeon

NO ↓

BACK PAIN DIAGNOSTIC TRIAGE

CONCERN

ACUTE LOW-BACK PAIN

Onset age 20-55 years

Lumbosacral, buttocks and thighs

"Above knee"

Mechanical pain

Patient well

90% recover from acute attack in 6 weeks

GUIDELINE APPLIES

SERIOUS

NERVE ROOT PROBLEM

Unilateral leg pain > LBP

Radiates to foot or toes

Numbness and Anaesthesia in same distribution

SLR reproduces leg pain

Localised neurology

50% recover from acute attacks in 6 weeks

VERY SERIOUS

RED FLAGS

Infection
- fever, immunosuppression, penetrating wound

Fracture
- significant trauma
- Minor trauma (if >50 years, osteoporosis)

Tumor
- Past history of malignancy
- Age >50 years
- Unexplained weight loss
- Pain at rest

Aortic aneurysm
- Absence of aggravating features

Red Flags ↓ SPECIALIST REFERRAL

Nerve Root Problem ↓ Is there a severe or aggressive motor weakness?

- YES → SPECIALIST REFERRAL
- NO → GP TREATMENT

Guideline applies ↓

GP TREATMENT 4-6 WEEKS

Provide reassurance:
- nothing dangerous
- expect recovery soon

No need for x-ray

Give advice to stay active (including activity log)

Give patient handout

Symptomatic measures if required
- simple analgesics, local heat

↓

Establish time for review

↓

Is pain improving satisfactorily?

- YES → Continued GP treatment
- NO → Check for Red Flags

Check for Red Flags

- YES → SPECIALIST REFERRAL
- NO → GP REVIEW

GP REVIEW

Was management plan implemented?

Explore issues and concerns

Consider referral

→ Establish time for review

12. Activity Log

Management of Low Back Pain in General Practice

Name: ____

Activity Log Sheet (please tick whether you were able to do your normal activities or not)

Date	Normal activities done	Unable to do normal activities	Comments

Staying active with back pain

So, what does 'staying active' really mean?

~~~~~~

Keep it moving

Keeping your back moving stops the working parts from seizing up. It may hurt a bit at first, but it does not do any damage - hurt is not the same as harm. It's worth working through any initial discomfort - because you'll get back to normal that much quicker.

You don't have to do special exercises. Simply continue to do your ordinary activities as normally as possible. Being fit and active will help you get better faster and prevent more back trouble later.

Strike a balance

Of course, you may need to take it a little easier or move a bit more carefully at first. But don't stop altogether. You can still do most normal activities without putting too much strain on your back - just use common sense!

What about work?

Some tasks may be more difficult when your back is sore, but back pain is not usually caused by work. Work is good for physical and mental health. So staying active and getting on with your life means staying at work or returning to work as soon as possible. You don't have to wait till the pain is 100% gone. In fact, getting back to work can help you recover faster. And don't be afraid to ask colleagues for help if you need to.

Get on with your life

You know that activity is good for your health - it's just the same for your back. The most important thing is for you to get on with your life. You really can help yourself.

~~~~~~

- Don't sit or stand in one position for too long - change position often.
- Get up and walk about to avoid stiffening up.
- Take breaks when driving.
- Some things may take a little longer or you may need to change how you do them.
- Pain killers may help you get going.
- Walking and swimming are good forms of regular exercise.

~~~~~~
~~~~~~

13. Patient handout

Number 1

Acute Low Back Pain

➔ *A partnership approach to pain management*

> What Is Acute Low Back Pain?

Acute low back pain is pain felt in the lower back that lasts for a short time (i.e. less than three months).

Most people have pain in their low back at some stage in their lives. In most cases, it will get better in several weeks; however, this varies from person to person. Acute low back pain may happen again over time.

> What Causes Acute Low Back Pain?

In around 95% of cases it is not possible to pinpoint the cause of the pain. However, it is not necessary to know the specific cause in order to manage the pain effectively.

It is rare for the pain to be caused by a serious medical problem.

> What Should I Do When I Have Acute Low Back Pain?

If your pain bothers you, it is important to see your health practitioner, to work with them to manage your pain, and to stay active.

1 *See your health practitioner*

A history and a physical examination are needed to assess for any serious medical conditions that may be associated with your pain, although these are rare.

Your practitioner can provide you with information about your pain once they have assessed you. Ask for an explanation if unfamiliar terms are used. Sometimes a diagram can be useful.

Additional investigations, such as xrays and blood tests, are not needed in the majority of cases of acute low back pain. They do not help with your pain or your ability to move your back.

It is normal to worry about the cause of your pain and the impact it may have on you. Talking to your health practitioner about your concerns can be helpful. You will usually find there is no serious cause and that there are ways to relieve your symptoms.

MAIN MESSAGES

- **Work with your health practitioner to manage your pain and address your concerns**
- **Stay active**

WHAT THE RESEARCH SAYS

A panel of experts recently reviewed the scientific studies on the effectiveness of treatments for acute low back pain and found that not all treatments have been studied in detail.

The findings of this review are published in the report *Evidence-Based Management of Acute Musculoskeletal Pain* available at www.nhmrc.gov.au. The results are summarised below.

Effective
Measures that are effective for relieving acute low back pain are: staying active (relieves pain better than resting in bed), having written information (it is helpful to discuss written information with your health practitioner) and heat wrap therapy (a treatment not routinely available in Australia).

Mixed results*
There are mixed results from scientific studies on the use of muscle relaxants, anti-inflammatory drugs (NSAIDs) and spinal manipulation. Some studies show these measures relieve acute low back pain and some do not.

Inconclusive*
Studies on acupuncture, back exercises, back schools, bed rest, cognitive behavioural therapy, injection therapy and topical treatments for acute low back pain have not tested these treatments against placebo.

No studies done*
There are no studies that have looked at: pain-relieving medication (analgesics), electromyographic biofeedback, lumbar supports, massage, multi-disciplinary rehabilitation in the workplace, traction and TENS for the treatment of acute low back pain.

*** It is important to note that these findings do not mean that these measures will not help you; they indicate that more research is needed.**

Australian Government
National Health and Medical Research Council

2 Work with your health practitioner to manage your pain

The goal is to help you find ways to manage your pain and return to your usual activities.

Most people find that their low back pain settles down over a short period of time as healing occurs. Pain-relieving measures may help you cope with your symptoms while nature takes its course.

There is a range of pain-relieving measures available. While there are few scientific studies proving their effectiveness, this does not mean that a particular measure will not help you (see What the Research Says).

When considering what measures to use for your pain, it is helpful to discuss the following points with your health practitioner:

- Your pain level and your concerns
- What measures are available to relieve acute low back pain (what they involve, how they work, their benefits and risks, their effectiveness)
- Your need for additional information

3 Stay active

Your pain may make it difficult to carry out your usual activities, and you may even want to rest completely.

However, it is important to resume normal activities as soon as possible. Staying active helps to prevent long-term problems.

You may need to use pain-relieving measures to help you gradually return to your usual activity level. If you are working, a programme of selected duties or reduced hours of work may be needed. This applies to work at home as well.

Follow-up visits

It is important to maintain contact with your health practitioner.

If the pain is not settling down or is getting worse, you may need further assessment.

Follow-up visits provide you with an opportunity to obtain more information. If you have any questions to ask your health practitioner, write them down and discuss them at your next visit.

The content of this information sheet is based on: Australian Acute Musculoskeletal Pain Guidelines Group (2003). *Evidence-Based Management of Acute Musculoskeletal Pain*, available at www.nhmrc.gov.au

The project was funded by the Department of Health and Ageing, Canberra.

Copyright © 2003 Australian Acute Musculoskeletal Pain Guidelines Group

ISBN 1864962275

Further copies of this information sheet are available from: www.nhmrc.gov.au/

Australian Government
National Health and Medical Research Council
